# GPU-Accelerated Signal Processing for Distributed Vibration Sensing Based on OVNA Method

**DOI:** 10.3390/s26113314

**Published:** 2026-05-23

**Authors:** Alessandro Meoli, Raffaele Vallifuoco, Agnese Coscetta, Luigi Zeni, Aldo Minardo

**Affiliations:** Department of Engineering, University of Campania Luigi Vanvitelli, Via Roma 29, 81031 Aversa, Italy; alessandro.meoli@unicampania.it (A.M.); raffaele.vallifuoco@unicampania.it (R.V.); agnese.coscetta@unicampania.it (A.C.); luigi.zeni@unicampania.it (L.Z.)

**Keywords:** OVNA, distributed optical fiber sensors, real-time signal processing

## Abstract

Distributed vibration sensing based on optical vector network analysis (OVNA) is a promising technique for measuring dynamic perturbations in optical fibers, but its practical use is limited by the high computational cost of short-time Fourier transform (STFT) and cross-correlation stages. In this work, we present a GPU-accelerated signal processing pipeline, together with an optimization strategy based on dataflow reduction, mixed-precision arithmetic, and hardware-aware tuning. The proposed implementation reduces the processing time for 200 sweeps from 64.7 s on a single-core CPU to 0.199 s on a modern GPU, while preserving the final shift results, with zero mismatches over 199,199 measurement points. Benchmarking across three GPU generations further shows that STFT benefits more from large on-chip cache resources, whereas cross-correlation scales more closely with memory bandwidth. These results suggest that modern GPUs can significantly reduce the computational burden of OVNA, as well as other distributed sensing methods with a similar processing flow, enabling kHz-rate aggregate throughput from batched processing, supporting real-time-oriented operation on modern GPUs.

## 1. Introduction

Distributed optical fiber sensors (DOFSs) have become important tools for structural health monitoring, seismic detection, and industrial monitoring applications [1,2]. In dynamic sensing applications, the most promising DOFS technologies rely on Rayleigh backscattering to detect and quantify small dynamic variations in the optical path length induced by external mechanical or thermal perturbations. Several measurement approaches have been proposed, operating either in the time domain, such as the phase-sensitive optical time-domain reflectometry (φ-OTDR) [3] and its pulse-compression variants [4], or in the frequency domain, such as optical frequency-domain reflectometry (OFDR) [5]. Time-domain approaches are usually preferred for long-range, fast acquisition measurements, while frequency-domain approaches are most suitable in applications requiring high spatial resolution. Recently, Loayssa et al. introduced a distributed vibration sensing technique based on optical vector network analysis (OVNA) [6]. In this approach, the reflective optical frequency-domain transfer function of the fiber under test is measured and converted, through short-time Fourier transform (STFT), into a sequence of frequency-dependent bandpass optical time-domain impulse responses. Such responses can be rearranged to extract a Rayleigh spectral signature at each sensing location. The Rayleigh signature provides fully linear and quantitative measurements of local strain or temperature variations, that are inherently free from the signal fading issue commonly affecting phase-measurement-based methods. Note that, while the OVNA originally proposed in Ref. [6] relied on a vector network analyzer (VNA) for data acquisition, the Rayleigh spectral signature can be equally extracted from optical time-domain measurements by preliminarily applying a fast Fourier transform (FFT) to the acquired signal [7].

Despite its advantages, the OVNA method suffers from a computationally demanding signal processing chain: each measured optical transfer function *H*(*ν*) must be processed through an STFT to obtain frequency-resolved impulse responses, followed by spectral reordering and sideband merging to reconstruct the Rayleigh signature matrix [6]. Consecutive measurements are then cross-correlated (as in conventional OFDR schemes), to extract the local frequency shift along the fiber, which encodes the applied perturbation. For typical processing parameters (*N* = 6000 frequency points, 200 sweeps, and about 1000 fiber positions), the reference implementation requires approximately 65 s on a single CPU core. This processing time is much longer than the acquisition time, which can be reduced to tens of milliseconds per sweep with an optimized RF demodulation subsystem, making computation the main obstacle to real-time-oriented operation [6,7].

**Figure 1:**
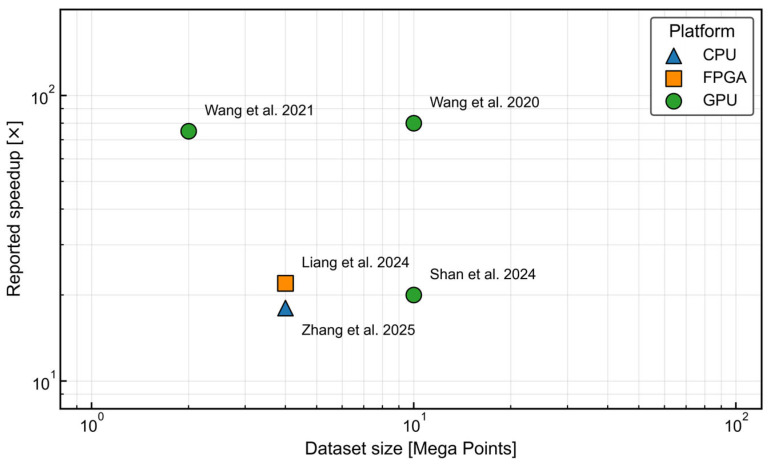
Comparison of reported acceleration by platform and dataset size. Speedup values are extracted from the cited literature. Baselines and datasets differ; results should be interpreted as indicative [8,9,10,11,12].

The parallel computing in the graphics processor unit (GPU) can significantly accelerate the data processing rate, because it contains many parallel data processing steps. In fact, GPU acceleration has been applied to address analogous bottlenecks in related optical sensing modalities, motivating its investigation for OVNA. In OFDR, Wang et al. [8] reported an ≈81× speedup, enabling 60 Hz continuous operation over a 200 m sensor through CUDA kernel tuning and cuFFT; Shan et al. [9] achieved a 21× latency reduction for multicore fiber shape sensing on an RTX 3090. A similar gain has been reported by Wang et al. in distributed acoustic sensing based on a linear frequency modulation pulse [10]. FPGA-based approaches reach comparable speedup factors [11], but at the cost of HDL development effort and on-chip memory constraints. Algorithmic improvements on CPU, such as the enhanced Buneman frequency estimator [12], yield more modest gains of ≈17×. These results are summarized in Figure 1.

Accelerating the OVNA pipeline further poses challenges that distinguish it from simpler GPU porting tasks. The STFT stage generates large intermediate arrays that must subsequently go through spectral reordering, sideband merging, and spatial selection before region of interest extraction. This is a sequence of dependent memory operations that, if left unoptimized, can jeopardize the gains obtained in the transform stage itself. The cross-correlation stage involves many independent pairwise operations on real-valued data, whose performance is sensitive to cache residency rather than raw floating-point throughput. Thus, a straightforward GPU port may still leave significant performance optimization unexploited due to memory-bound postprocessing bottlenecks.

In this work, we present a GPU acceleration of the complete OVNA signal processing pipeline. The approach is validated on the experimental dataset reported in [6] (200 sweeps, 6000 frequency points, 115 m fiber) and achieves an end-to-end speedup of up to 325× while preserving the final shift results. The main contributions of this paper are:

- A fused spectral-reorder and fiber-selection index that combines fftshift, circular shift, flip, and spatial selection into a single gather operation, reducing both postprocessing data volume and execution time.
- A mixed-precision strategy, based on float32 STFT and float64 cross-correlation, that preserves the extracted shift results over the full dataset.
- A hardware-aware tuning of batch size and numerical precision based on queried GPU properties, including memory bandwidth, L2 cache size, and floating-point performance ratios.
- A cross-architecture benchmark across three NVIDIA GPU generations, showing that STFT benefits more strongly from large on-chip cache resources, whereas cross-correlation scales more closely with memory bandwidth.

While each of these primitives (batched FFT, cuFFT-based correlation, mixed-precision arithmetic) has been individually exploited in prior GPU-accelerated distributed sensing efforts, the present work integrates them with two contributions specific to the OVNA application: a fused spectral-reorder index that exploits the dual-sideband + ROI structure of the OVNA pipeline (Section 3.5), and a runtime hardware-aware autotuner (Section 3.7) that adapts execution parameters to GPU properties without manual configuration.

The remainder of this paper is organized as follows. Section 2 reviews the OVNA principle and signal processing chain. Section 3 describes the GPU acceleration strategy. Section 4 presents the mixed-precision analysis. Section 5 reports the benchmark results across multiple GPU architectures. Section 6 discusses the optimization approaches that were investigated but did not provide measurable benefit and in Section 7 conclusions are drafted.

## 2. OVNA Principle and Computational Bottleneck

**Figure 2:**
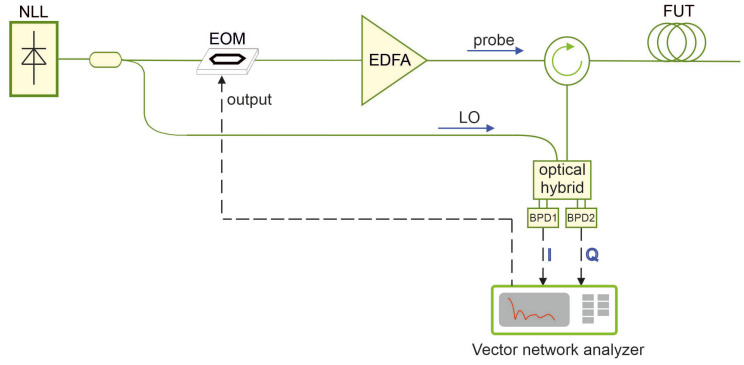
Schematic representation of the OVNA setup. NLL: narrow-linewidth laser; EDFA: erbium-doped fiber amplifier; EOM: electro-optic modulator; LO: local oscillator: BPD: balanced photodetector; FUT: fiber under testing.

We briefly recall the OVNA principle and signal processing following the formulation in [6]. In OVNA, the optical transfer function of the fiber under test (FUT) is acquired using the scheme shown in Figure 2. In brief, the light from a narrow-linewidth laser (NLL) emitting at $f_{0}$, is split into a local oscillator (LO) and a probe signal. The latter is a double sideband (DSB) modulated at frequency $f_{m}$ through an electro-optic modulator biased at its null point and driven by the RF output of a VNA. The Rayleigh backscatter produced by the probe during its propagation along the FUT, is mixed with the LO through a 90° optical hybrid, which works in conjunction with the VNA and two balanced photodetectors to extract the in-phase (I) and quadrature (Q) components of the backscattered light. Finally, these two components are properly combined to reconstruct the two-sided optical transfer function H(ν) at frequencies $\nu_{i}=f_{0}\pm f_{m,i}$, where $f_{m,i}$ (with i=0,…N−1) are the modulation frequencies swept by the VNA. The extension and step of such frequency sweep determine the spatial resolution and the alias-free measurement range, respectively [6].

### 2.1. STFT Decomposition

To extract the Rayleigh spectral signature related to each fiber position, the measured transfer function is decomposed using a sliding spectral window. As shown in [6], the frequency-resolved impulse responses $h_{k}(m)$ are obtained by calculating the STFT of H(n) with a window length $N_{S}$ and hop size Δk, and applying a time-inversion to the resultant $h_{k}(m)$. In practical cases, the minimum modulation frequency $f_{m,0}$ may differ from the frequency step ${∆f}_{m}$ chosen for the VNA sweep, which means that a frequency gap occurs at the center of the recovered H(n) [6]. In these cases, two separate STFTs (one per sideband) must be calculated. The number of impulse responses $h_{k}\left(m\right)$ retrieved from each STFT is given by $K=\left(N-N_{S}\right)/∆k+1$. Furthermore, the spatial granularity of the impulse responses can be arbitrarily increased to $N_{FFT}$, by using zero-padding. Therefore, each STFT provides K impulse responses, each composed by $N_{FFT}$ spatial samples. According to the OVNA principle, the impulse responses are rearranged to form $N_{FFT}$ Rayleigh signatures (one per spatial position), each one composed by K frequency samples [6]. In case of two separate STFT calculations, the Rayleigh signatures from each sideband are finally merged (with appropriate conjugation for the lower sideband), resulting in 2K frequency samples per position.

### 2.2. Spectral Postprocessing

**Figure 3:**
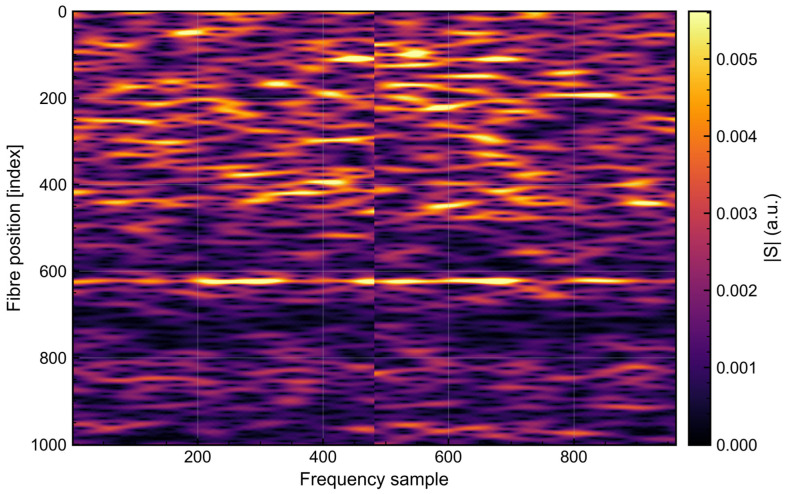
Merged STFT output (amplitude) for a single measurement sweep, showing the frequency-dependent Rayleigh backscatter pattern across 1001 fiber positions. The left half corresponds to the conjugated lower sideband and the right half to the upper sideband. The discontinuity in the middle is due to the spectral gap between $f_{0}-500\;MHz$ and $f_{0}+500\;MHz$.

The raw STFT output requires several reordering operations to produce the correctly centered and oriented impulse response. For each sideband, the STFT output is: recentered via fftshift, circularly shifted to align the spatial origin and flipped to obtain the correct spatial ordering. The two sidebands are then concatenated (with appropriate conjugation for the lower sideband) to form the merged Rayleigh signature matrix of dimensions $N_{FFT}\times 2K$. Finally, a spatial region of interest (ROI) containing $N_{ROI}$ fiber locations is selected, and the modulus of the Rayleigh signatures is computed on that ROI. Figure 3 shows a representative merged STFT output for a single sweep of the 115 m fiber considered in [6], displaying the characteristic Rayleigh backscatter pattern over a ROI containing 1001 fiber locations.

### 2.3. Cross-Correlation for Frequency Shift Extraction

The frequency shift ${\Delta f}_{k}$ in the Rayleigh signature between consecutive measurement sweeps is extracted via FFT-based cross-correlation. For each pair of successive sweeps (i, i + 1), the merged amplitude matrices are cross-correlated along the frequency axis at each fiber position. The cross-correlation length is the next power of two exceeding 2 × 2K − 1. The peak position, determined via argmax, gives the delay in bins, which is converted to a frequency shift via $∆f=dbins\times ∆k\times {∆f}_{m}$. For $N_{sw}$ sweeps, this produces a shift array of size ($N_{FFT}$, $N_{sw}-1$) encoding the spatially resolved frequency shift profile.

### 2.4. Computational Profile

**Figure 4:**
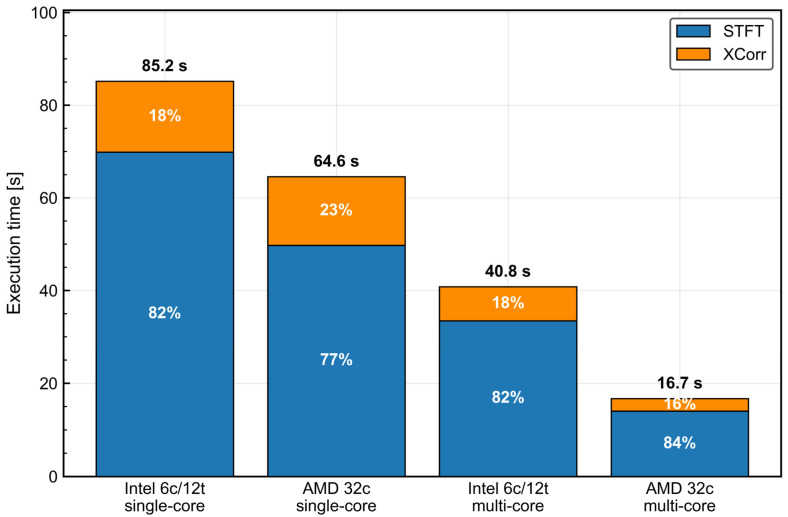
CPU baseline execution time breakdown across two systems and four configurations. The STFT phase (orange) dominates in all cases. Multicore parallelization provides 2.1× (Intel 12 workers) to 3.9× (AMD 32 workers) speedup, but the best CPU configuration (16.7 s) remains far from real-time requirements, motivating GPU acceleration.

Table 1 summarizes the pipeline parameters. The reference single-core CPU implementation (scipy/numpy, Python 3.13) completes in 64.7 s on the AMD platform. Multicore parallelization across all available cores reduces total time to 16.7 s (AMD, 32 workers). In all configurations, the STFT phase accounts for 77 to 84% of total time, with the remaining 16 to 23% spent on cross-correlation, far exceeding the target acquisition time of tens of milliseconds, see Figure 4.

## 3. GPU Acceleration Strategy

The GPU acceleration strategy follows a profiling-driven approach: each optimization is applied incrementally, with numerical validation against the CPU reference before proceeding. Table 2 summarizes the hardware platforms used for benchmarking. All GPU code uses CuPy, an open-source framework for GPU-accelerated computing with Python [13], as the acceleration framework. The three selected GPUs are representative of three different NVIDIA generations, from the low-cost, low-performance GTX 1050 Ti, up to the professional-grade RTX PRO 6000.

### 3.1. Baseline GPU Port

The initial GPU implementation is a direct translation of the CPU pipeline: each sweep is processed independently with the cupy.fft.fft function replacing scipy.signal.stft, and each cross-correlation pair is computed sequentially. This baseline achieves 0.324 s total on the Blackwell GPU (200× speedup), 0.510 s on the 3090 Ti (127×), and 5.127 s on the 1050 Ti (12.6×). All speedup figures in this paper are normalized to the AMD 32-core single-thread CPU reference (64.7 s) unless otherwise noted.

### 3.2. Data Residency and Precomputation

The first major optimization eliminates redundant data transfers and kernel launches. The full upper and lower sideband arrays are uploaded to the GPU memory once at initialization. Frame indices, fiber indices, and the spectral reordering map are precomputed on the GPU. Most significantly, the three-step spectral reordering (fftshift, circular shift by s bins, flip) is collapsed into a single precomputed index array: $remap[i]\;=\;(N_{FFT}/2-\;s\;+\;N_{FFT}\;-1\;-\;i)\;mod\;N_{FFT}$, where s is the circular shift value. This eliminates two kernel launches per sweep and reduces the reordering to a single fancy-indexing gather.

### 3.3. Batched Cross-Correlation

The cross-correlation inputs are real-valued, but the initial implementation uses full complex FFTs. Replacing fft/ifft with rfft/irfft computes only the positive half of the spectrum: ($N_{cc}$/2 + 1) complex elements instead of $N_{cc}$. This yields a 16% GPU reduction and 32% CPU reduction in cross-correlation time. The per-pair loop is replaced by a batched execution. All sweep pairs are stacked into a 3D array and the rfft, conjugate multiplication, irfft, and argmax are issued as single-batched operations. A custom argmax kernel combines peak detection and conversion of the correlation peak index into a signed delay in a single step. It reads directly the Fortran-ordered output of cuFFT using strides, so no ascontiguousarray copy is needed. This reduces cross-correlation time from 133 ms to 111 ms.

### 3.4. Batched STFT Execution

Rather than processing each sweep independently, all sweeps are batched into a single cupy.fft.fft call. The framing operation constructs a 3D array of shape (*B*, $N_{FFT}$, K) where B is the autotuned batch size (i.e., the number of sweeps contained in a single batch). On the Blackwell GPU (95.6 GB VRAM), all 200 sweeps fit in a single batch (B =200) while on the 3090 Ti (24 GB), the autotune selects B =38; finally on the 1050 Ti (4 GB), VRAM constraints force B =1, effectively disabling batching while still benefiting from all other GPU optimizations.

### 3.5. Fused Fiber Selection in Postprocessing

Profiling of the batched pipeline revealed that the postprocessing stage was the dominant bottleneck, as summarized in Table 3. The root cause is that all operations prior to fiber selection operate on the full 8192-row arrays, discarding 87.8% of the data only at the final step. The solution is to precompute a fused index that performs the spectral reordering and fiber selection in a single gather: remap_fiber[j] = remap[ind_fiber[j]], j = 0, …, nfiber − 1. This transforms the gathering from 8192 to 1001 rows, reducing intermediate memory from 26.8 GB to 3.1 GB (8.7×) and postprocessing time from 110 ms to 7 ms (15.7×). After this optimization, the cuFFT call (31 ms) becomes the dominant cost, which represents the theoretical lower bound for an FFT-dominated pipeline, the theoretically optimal configuration.

At the full pipeline level, the cumulative breakdown reported in Section 5.3 (Table 9) shows that the fused fiber-selection index is the second-largest single contributor to the overall GPU-batch speedup, after data residency on the GPU.

### 3.6. Multicore CPU Baseline

For comparison, a multicore CPU implementation parallelizes independent sweeps (STFT) and pairs (cross-correlation) using ThreadPoolExecutor across all 32 logical cores. Correctness relies on non-overlapping write regions for each thread. The scipy.signal.stft function was replaced with manual vectorized framing to release the Global Interpreter Lock (GIL).

### 3.7. Hardware-Aware Autotuning

A GPUInfo dataclass queries GPU hardware properties via CuPy at initialization and autotunes the STFT batch size, subject to SM saturation (at least 4 FFTs per SM), memory budget constraints (accounting for inputs, STFT output, cuFFT temporaries estimated at 2.5×, and postprocessing intermediates) and the STFT precision (float32 when the FP32/FP64 ratio exceeds 2, which includes all modern NVIDIA architectures). This enables a single codebase to adapt across GPU generations without manual tuning.

## 4. Mixed-Precision Analysis

Modern GPU architecture exhibits large performance asymmetries between single- and double-precision arithmetic. The Blackwell and 3090 Ti GPUs have FP32/FP64 ratios of 64:1, while the 1050 Ti has 32:1; in all cases, measured cupy.fft.fft throughput on complex64 data is several times faster than on complex128. This motivates a mixed-precision approach, but validation is essential since the cross-correlation argmax calculation is sensitive to small differences in the correlation peak. We performed a validation across all 200 sweeps × 1001 fiber positions (199,199 shift values). Table 4 reports the results, where mismatches are evaluated in comparison with a complex128 implementation.

The float32 STFT + float64 cross-correlation produces zero mismatches—complete bit-exact agreement. Full float32 introduces 12 mismatched shift values (0.006%), occurring at fiber positions where the cross-correlation peak has nearly equal amplitude at adjacent bins. The adopted strategy yields a 2.5× speedup in the STFT phase with zero impact on accuracy. Note that the STFT normalization differs between CPU (scipy convention: divide by window sum) and GPU (divide by sampling frequency); since the cross-correlation argmax is scale-invariant, this does not affect the output shift values.

The mixed-precision claim made in this work is limited to preservation of the final extracted shift values on the dataset considered here, while a broader numerical equivalence analysis of intermediate correlation amplitudes is left for future work.

## 5. Benchmark Results

In this section, we provide benchmark results for the tested hardware platforms and computing operations (STFT + XCorr). All timings report the median over five repetitions, with GPU phases timed using CUDA events, and CPU phases using high-resolution wall-clock timers. A warmup iteration precedes timed measurements to ensure that cuFFT plan creation does not affect results. All tiers produce numerically identical output (max|Δshift| = 0.00, sign agreement = 100%).

### 5.1. GPU Benchmarking

Table 5, Table 6 and Table 7 report the results of our analysis for the RTX PRO 6000 Blackwell, the RTX 3090Ti and the GTX 1050Ti, respectively. Note that the 1050 Ti represents a VRAM-constrained scenario: with 4 GB, the autotuner selects B =1, making batching ineffective. The system also uses a different CPU (Intel 6-core/12-thread vs. AMD 32-core), resulting in a slower baseline (85.4 s vs. 64.7 s).

**Figure 5:**
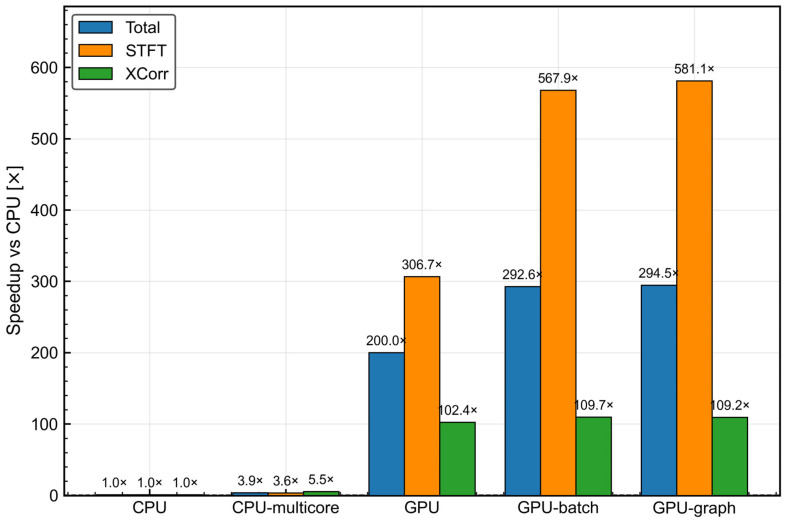
Per-tier speedup over CPU baseline (RTX PRO 6000 Blackwell GPU). Blue: total; orange: STFT phase; green: cross-correlation phase.

**Figure 6:**
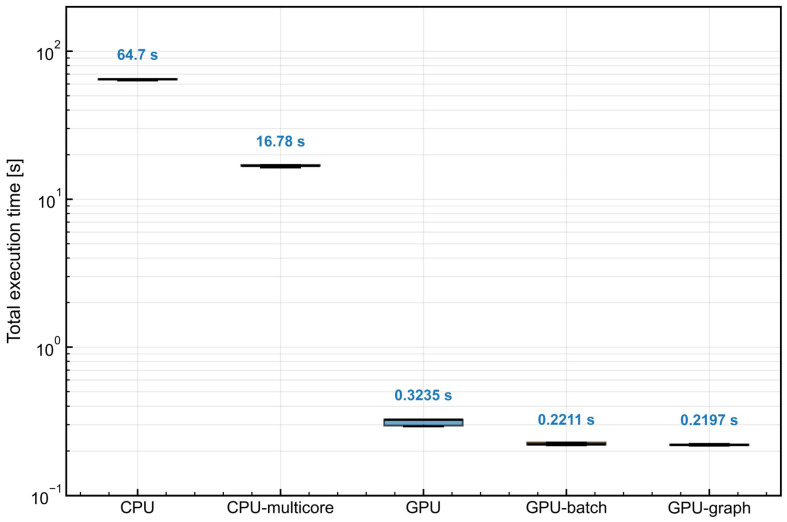
Timing distribution across five repetitions for each tier (Blackwell GPU). GPU tiers exhibit very low variance (IQR < 0.01 s), demonstrating deterministic execution.

We summarize in Figure 5 the speedup factors obtained with the most performant GPU (the RTX PRO 6000 Blackwell), highlighting the greater effectiveness of batching and mixed precision on the STFT phase, compared to the cross-correlation phase. Finally, Figure 6 reports the timing distribution of the five repetitions of the tests performed on the same GPU, with speedup figures normalized to the common AMD 32-core single-thread reference.

### 5.2. Cross-Architecture Comparison

Table 8 compares the GPU performance across the three tested GPU architectures. To ensure a fair comparison independent of the host CPU, all speedup figures are normalized to the same reference: the AMD 32-core single-thread CPU baseline, measured on the Blackwell system. This avoids inflating the 1050 Ti’s apparent speedup, whose Intel 6-core host is 32% slower. The cross-architecture comparison is conducted at the GPU-batch tier, which was benchmarked on all three platforms. This to ensure a fair hardware comparison. A further batched cross-correlation optimization was applied to the Blackwell GPU only (the primary development target) yielding an additional reduction from 0.221 s to 0.199 s (Table 5).

As regards the cross-correlation phase, the Blackwell achieves 0.133 s versus the 1050 Ti’s 2.043 s (15.4× ratio), which is close to the 16× theoretical memory bandwidth ratio (1792 vs. 112 GB/s). The 3090 Ti sits at 0.305 s, giving Blackwell/3090 Ti = 2.29× (vs. 1.78× bandwidth ratio) and 3090 Ti/1050 Ti = 6.7× (vs. 9× bandwidth ratio). Therefore, the cross-correlation results indicate a speedup factor scaling linearly with the memory bandwidth.

As regards the STFT phase, the results indicate the following speedup factors: Blackwell/1050 Ti = 35.0×, Blackwell/3090 Ti = 1.90×, and 3090 Ti/1050 Ti = 18.46×, revealing a more-than-linear dependence from the memory bandwidth. This not-strictly-memory-bound behavior may be attributed to the influence of the L2 cache memory, which is only 1 MB for the 1050 Ti, thus insufficient to perform batching.

**Figure 7:**
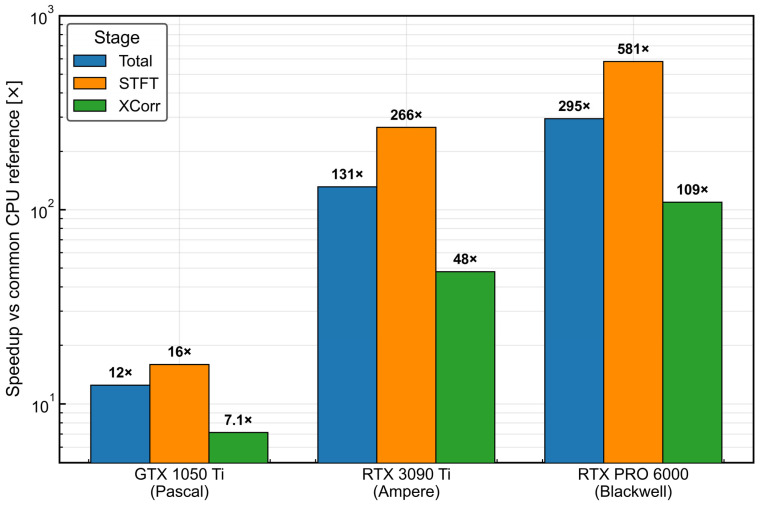
Cross-architecture GPU speedup normalized to a common AMD 32-core single-thread CPU baseline (64.7 s). The STFT phase (orange) scales linearly from batching and SM-count benefits, while the cross-correlation (green) is limited by L2 cache capacity on smaller GPUs.

**Figure 8:**
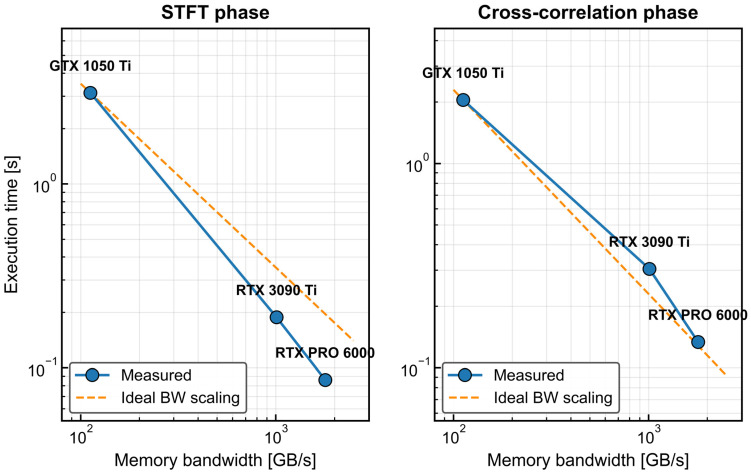
Execution time versus memory bandwidth for the STFT and cross-correlation phases across three GPU architectures.

These effects are visualized in Figure 7 where the bar chart shows the normalized speedup across all three GPUs, and in Figure 8 where the log–log scaling plot compares the measured performance against ideal bandwidth scaling, clearly revealing the L2 miss penalty at the 3090 Ti and the linear STFT scaling from batching.

### 5.3. Optimization Progression

Table 9 traces the cumulative effect of each optimization step on the GPU-batch tier (Blackwell), illustrating the contribution of each algorithmic improvement. The table reveals that the largest improvement results from data residency, while subsequent refinements have a minor impact.

### 5.4. Experimental Validation

**Figure 9:**
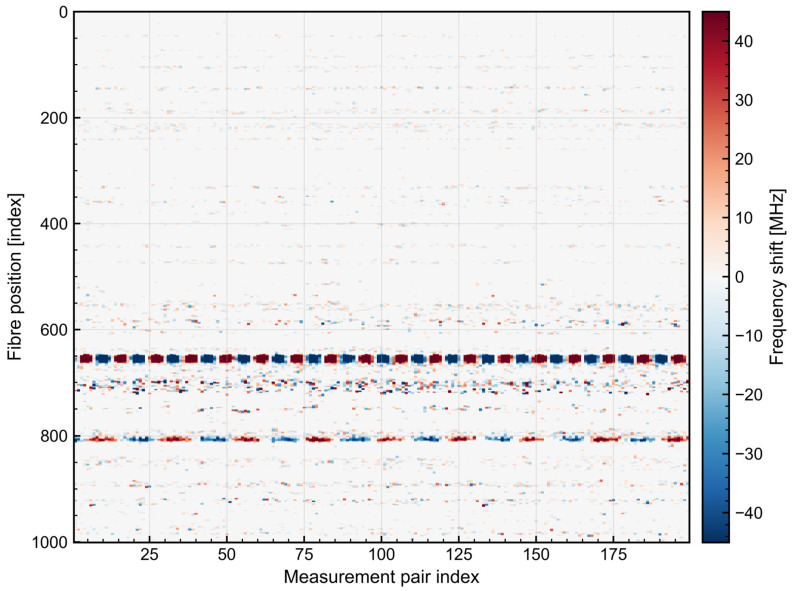
Frequency shift map (Hz) showing the spatially resolved Rayleigh signature shifts across 199 sweep pairs. Two localized excitations are visible near fiber positions 650 (Peltier cell) and 800 (piezoelectric cylinder), consistent with the experimental setup in [6].

Figure 9 shows the frequency shift map computed from the experimental data of [6], representing the spatially resolved Rayleigh signature shifts across 199 consecutive sweep pairs and 1001 fiber positions. Two localized excitation regions are clearly visible: a thermal perturbation (Peltier cell) near fiber position 650 and a piezoelectric excitation near position 800.

**Figure 10:**
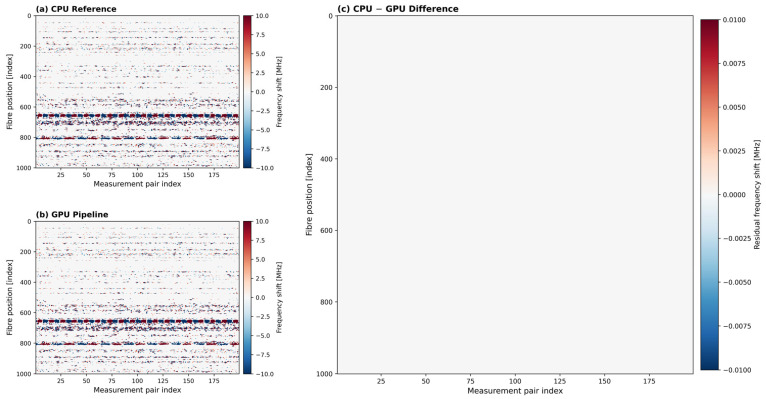
CPU vs. GPU-graph validation. Frequency shift maps: (**a**) CPU, (**b**) GPU, (**c**) GPU-CPU.

Figure 10 compares the CPU reference and GPU-graph outputs on the full experimental dataset. For the mixed-precision configuration adopted in the final pipeline (float32 STFT and float64 cross-correlation), the extracted shift maps are identical to the CPU reference over all 199,199 evaluated points. The systematic exploration of independent physical acquisition conditions, beyond the dataset of Ref. [6] used here, is left to future work.

### 5.5. Implication for Real-Time-Oriented Operations

The GPU-batch processing time of 0.199 s for 200 sweeps corresponds to an average throughput equivalent to ≈1.0 ms per sweep. On the 3090 Ti, the corresponding value is ≈2.4 ms per sweep, and on the 1050 Ti it is ≈25.6 ms per sweep. Compared with the estimated OVNA sweep acquisition time for the parameters of [6], which remains in the tens-of-milliseconds range, these results indicate that signal processing is no longer the dominant bottleneck on the tested platforms (except for the 1050 Ti GPU).

As an additional check of streaming-mode behavior, per-chunk latency was measured by splitting the dataset into chunks of M = {10, 20, 50} consecutive sweeps and running the GPU pipeline once per chunk; on the tested GPU, the per-chunk latency was found compatible with a 100 Hz streaming target across all M values, while higher per-chunk rates remain limited by the per-call setup overhead. Demonstrating live acquisition with real-time sustained streaming infrastructure is left to future work.

These measurements do not constitute a demonstration of a fully streaming real-time system, since they were obtained on pre-acquired datasets using batched execution. However, they do show that the achieved computational throughput is compatible with real-time-oriented operation, particularly on modern GPUs.

### 5.6. Three-Parameter Empirical Performance Model

The measurements collected across three GPU architectures, spanning a 16× range in memory bandwidth, are interpreted using a simple empirical model with three fit parameters. Since both pipeline stages are predominantly memory-bound, the natural scaling variable is the ratio of data volume to effective memory throughput.

For the STFT phase, the workload consists of $N_{sw}\cdot K$ batched FFTs of size $N_{FFT}$ in complex64, where $K=\frac{N-N_{S}}{∆k}+1$ accounts for the sliding window overlap. The total data volume, weighted by the FFT algorithmic complexity, is [14]:

(1) $$W_{S}=N_{sw}\cdot K\cdot N_{FFT}\cdot {log}_{2}\left(N_{FFT}\right)\cdot s_{c64},$$

where $s_{c64}$ is the size (in bytes) of the FP64 data type (note that two FP32 data are required, for the real and imaginary parts). The effective throughput depends on the memory bandwidth (BW) and on how many FFTs can be processed simultaneously, which is limited by the smaller of the batch size *B* and the number of streaming multiprocessors $N_{SM}$. We model:

(2) $$t_{STFT}=\alpha_{S}\frac{W_{S}}{BW\cdot min{\left(B,N_{SM}\right)}^{\beta }}.$$

where the prefactor α_S_ accounts for cuFFT kernel overhead, SM occupancy limits, and the gap between peak and sustained bandwidth. The exponent *β* < 1 reflects the fact that doubling the SM count does not halve the execution time, as the memory subsystem progressively becomes the dominant constraint. Note that *t_STFT_* does not depend on *N_f_*, because the full FFT is computed on each sweep regardless of how many spatial positions are eventually selected. For the cross-correlation stage, each of the $N_{pairs}=\;N_{sw}-1$ sweep pairs undergo two rfft calls, a conjugate multiplication, an irfft, and an argmax, all in float64. The corresponding single-pass data volume is:

(3) $$D=N_{pairs}\cdot N_{ROI}\cdot N_{FFT}\cdot s_{c64}.$$

Because the pipeline involves multiple read/write passes over the data, the effective number of DRAM traversals is captured by a single coefficient $c_{0},$

(4) $$t_{XCorr}=c_{0}\frac{D}{BW}.$$

The parameter $c_{0},$ therefore, represents the effective number of full-volume DRAM passes required by the complete per-pair processing chain. Note that $t_{XCorr}$ grows linearly with $N_{ROI}$. For kilometer-scale fibers, this scaling suggests a substantial increase in cross-correlation time with respect to the present 1001-point case, making it the dominant stage.

An L2 cache dependent term was also initially considered, based on the ratio between the active working set $W_{act}=N_{ROI}\cdot N_{FFT}\cdot s_{c64}$ and the available L2 capacity. Although this extension was physically motivated, it produced only a marginal improvement in fit quality with the present three-point dataset. For this reason, the simpler formulation was retained, and the possible role of cache residency is interpreted qualitatively rather than claimed as an independently identified model component. However, for long (km-scale) sensing fibers, $W_{act}$ could grow well beyond the L2 cache of current GPUs, which indicates that cache residency and memory traffic are likely to become increasingly important at longer sensing ranges. Fitting Equations (2) and (4) simultaneously to the measurements (three GPUs × two stages) yields α_S_ = 4.07, β = 0.16, and c_0_ = 9.6.

**Figure 11:**
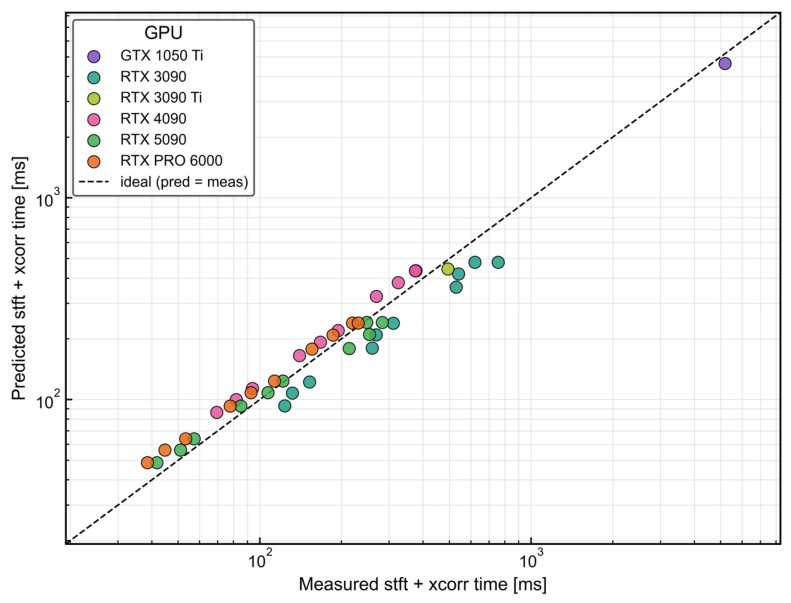
Predicted versus measured stft + xcorr execution time on the extended GPU dataset. Multiple markers per color correspond to a sensitivity sweep over the number of sweeps ∈ {50, 100, 200} and the number of fiber positions ∈ {500, 750, 1001} at a fixed number of frequency points.

The model reproduces the measured total times within 8.4% or better across the three tested architectures (see Figure 11 for a visual representation of fit quality). Its purpose is not to resolve all microarchitectural effects, but rather to capture the dominant scaling trends with a minimal number of parameters. The largest per-stage discrepancy occurs for the 3090 Ti cross-correlation (−18.7%), where the simple bandwidth scaling slightly overestimates the achieved throughput.

As a further consistency check, the model was fitted on two GPUs and evaluated on the held-out third. The resulting total-time errors are −20% for the 1050 Ti, −9% for the 3090 Ti, and −4% for the RTX PRO 6000. The relatively large error on the 1050 Ti is expected, since this GPU operates in a VRAM-constrained regime (B = 1), where the scaling behavior differs qualitatively from that of the higher-end devices. The smaller errors on the 3090 Ti and RTX PRO 6000 suggest that the model captures the dominant scaling trend in the regime most relevant to modern GPUs, while remaining too approximate for strong extrapolative claims.

To strengthen the validation of this model, we have further tested it over six GPUs, spanning four NVIDIA architectures (Pascal, Ampere, Ada, Blackwell) and a 16× memory-bandwidth range. A sensitivity sweep was collected by varying the number of sweeps *N_sw_* ∈ {50, 100, 200} and the number of fiber positions *N_ROI_* ∈ {500, 750, 1001} at fixed *N* = 6000 frequency points and *N_FFT_* = 8192, yielding nine workload configurations per GPU. Combined with the six full-data anchor points (one per GPU, at Nsw = 200, NROI = 1001), the resulting sensitivity dataset comprises 42 measurement records and exercises the two workload axes that dominate the model:

The empirical model has been re-fitted on the extended dataset, yielding α_S_ = 4.39, β = 0.10, c_0_ = 2.12 (Figure 11), with an in-sample mean absolute error of 17% and a maximum of 37%. The leave-one-out errors per GPU are −0.7% (RTX 5090), −10% (RTX 3090 Ti), −17% (GTX 1050 Ti), +22% (RTX PRO 6000), +25% (RTX 4090), and −32% (RTX 3090), with the larger errors corresponding to GPUs whose architecture is not represented in the training set. As a particularly informative controlled experiment, the RTX 5090 (consumer Blackwell) and the RTX PRO 6000 (workstation Blackwell) share the same GB202 silicon and identical 1792 GB/s memory bandwidth: the held-out 5090 prediction differs from the measurement by less than 1%, providing direct evidence that the model captures the dominant scaling within an architectural family. Across architectural boundaries, the residual leave-one-out error indicates that the simple three-parameter form is best interpreted as a qualitative scaling guide; the estimates of Table 10 should accordingly be read as indicative rather than predictive, with a larger uncertainty when extrapolated to architectures outside the training set.

Both stages operate well below the FP32 compute roofline, confirming the predominantly memory-bound regime. However, the float64 arithmetic used in the cross-correlation stage places the workload closer to the FP64 ridge point on consumer GPUs, suggesting that FP64 throughput may act as a colimiting factor.

A detailed kernel-level analysis with NVIDIA Nsight Compute would be required to further disambiguate these effects and is left for future work.

The above-described model can be used to estimate the approximate maximum fiber length sustainable at a given demodulation rate. Table 10 reports these indicative values for the RTX PRO 6000 Blackwell, where we assumed that all other measurement parameters are kept the same as the ones considered here.

Overall, these numbers suggest that, as the data processing is no longer the bottleneck, real-time-oriented OVNA demodulation at high rates becomes in principle feasible. However, this conclusion assumes that the rest of the system can sustain the same throughput. This aspect will be investigated in future work.

### 5.7. Generalization to Other Distributed Sensing Techniques

While the present paper is focused on the OVNA technique, the processing structure analyzed in this work is shared by other distributed sensing techniques that extract frequency shifts from swept or pulsed measurements [5,15,16]. Compared to the conventional OFDR based on swept-wavelength interferometry, the OVNA processing chain does not require any preliminary FFT of the acquired data. Apart from this, the rest of the processing is identical.

The three algorithmic optimizations presented here are not OVNA specific in their underlying rationale. The fused spectral-reorder index targets memory-bound postprocessing, the mixed-precision strategy (float32 STFT and float64 cross-correlation) provides a practical compromise for this class of pipelines, and the batch autotuner adapts the execution strategy to different GPU memory sizes without requiring code changes.

The quantitative validity of the present performance model outside the OVNA case should be considered as indicative. Different sensing techniques may alter FFT sizes, data layout, correlation depth, arithmetic precision, and intermediate memory traffic. Therefore, while the optimization principles are likely transferable, the model itself should be adapted and revalidated for each specific pipeline.

## 6. Approaches Investigated Without Relevant Improvement

Several optimization strategies, in addition to the ones reported here, have been implemented and benchmarked, but none of them provided measurable improvement.

Distributing pairs across multiple CUDA streams did not improve performance, indicating that the individual operations already saturated the available GPU resources. A custom CUDA argmax kernel achieved a strong per-call speedup in isolation, but this advantage disappeared in the full loop because CuPy’s asynchronous execution already overlaps the argmax of one pair with the FFT processing of the next.

CUDA graph capture was also investigated, but CuPy’s high-level memory-pool behavior caused address-reuse issues during replay, and in any case the expected benefit was limited because kernel launch overhead represented only a negligible fraction of the total runtime.

These negative results suggest that, for the present OVNA pipeline, memory traffic and execution overlap are more critical than further reduction in kernel launch overhead. Accordingly, future optimization efforts should focus on reducing data movement, improving data locality, and increasing the pipeline-level overlap between processing stages, rather than on further micro-optimizing already well-hidden kernel launches. In particular, architectures that reduce or eliminate explicit host-device data transfers by combining CPU and GPU in a single module with shared memory may offer a more promising path for further gains.

## 7. Conclusions

We have presented a GPU-accelerated signal processing pipeline for OVNA distributed sensing, validated on experimental data from a 115 m fiber under thermal and strain excitations. Through profiling-driven optimization, the end-to-end processing time was reduced by 325× on a Blackwell GPU normalized to a common single-core CPU reference. The output is numerically identical to the CPU baseline across all 199,199 measurement points. The principal algorithmic contribution is the fused spectral-reorder and fiber-selection index, a batched cross-correlation execution that eliminates pair kernel launch overhead and a mixed-precision strategy (float32 STFT, float64 cross-correlation) that provides speedup without sacrificing numerical accuracy.

A three-parameter analytical model, fitted on the three GPU architectures captures the scaling of both pipeline stages with memory bandwidth and parallelism. The model captures the dominant scaling trends across the tested architectures. The three algorithmic optimizations are likely transferable to any pipeline sharing the STFT plus cross-correlation structure.

We further believe that GPU-equipped optoelectronic interrogators may leverage GPU acceleration not only for strain demodulation as discussed in this paper, but even for real-time event classification via machine learning models [17].

Performance projections based on the model indicate that real-time-oriented OVNA is in principle achievable. Ongoing work focuses on the realization of an embedded platform that combines the NVIDIA Jetson AGX Thor [18] with an RFSoC-based [19] acquisition front end.

## Figures and Tables

**Table 1:** List of parameters adopted for GPU performance assessment.

Parameter	Value	Description
$f_{m,0}$	500	Minimum VNA modulation frequency (MHz)
$f_{m,N-1}$	3500	Maximum VNA modulation frequency (MHz)
${∆f}_{m}$	0.5	VNA modulation frequency step (MHz)
N	6000	Frequency points per sweep
$N_{sw}$	200	Number of measurement sweeps
$N_{S}$	1200	STFT window length
Δk	10	STFT hop size (samples)
$N_{FFT}$	8192	FFT zero-pad length
K	481	STFT frames per sweep
$N_{ROI}$	1001	Selected fiber positions (indices 7000–8000)
2K	962	Merged spectrum columns
$N_{cc}$	2048	Cross-correlation FFT length

**Table 2:** Benchmark system configurations.

Component	GPU0	GPU1	GPU2
GPU	RTX PRO 6000	RTX 3090 Ti	GTX 1050 Ti
Architecture	Blackwell	Ampere	Pascal
Number of streaming multiprocessors (SM)	188	84	6
VRAM	95.6 GB	24.0 GB	4.0 GB
Memory bandwidth BW (theor.)	1792 GB/s	1008 GB/s	112 GB/s
L2 cache	134 MB	6 MB	1 MB
Compute capability	12.0	8.6	6.1
FP32/FP64	64:1	64:1	32:1
CPU	AMD 32 cores	AMD 32 cores	Intel 6c/12t
CUDA version	13.0	13.0	12.9
OS/Python	Win 11/3.13	Win 11/3.13	Win 11/3.13

**Table 3:** STFT postprocessing profiling before and after fused fiber selection (executed on the RTX PRO 6000, *B* = 200).

Operation	Before [ms]	After [ms]	Improvement
Gather + window	3	3	—
Zero-pad + copy	5	5	—
cuFFT (8192-pt)	31	31	—
Remap gather	9	2	4.5×
Conj + reverse + concat	70	4	17.5×
Fiber select + abs + swap	51	—	eliminated
Abs + swapaxes	—	2	—

**Table 4:** Mixed-precision validation results.

Configuration	Mismatched Shifts	Maximum Error
float32 STFT + float64 xcorr	0 (0.000%)	0.00
Full float32 (STFT + xcorr)	12 (0.006%)	-

**Table 5:** Performances on RTX PRO 6000 Blackwell (median over 5 repetitions).

Tier	STFT [s]	XCorr [s]	Total [s]	Speedup	Max|Δ|
CPU	50.004	14.590	64.696	1.0×	—
CPU-multicore	14.060	2.679	16.783	3.9×	0.00
GPU per-sweep	0.163	0.142	0.324	200×	0.00
GPU-batch	0.088	0.133	0.221	293×	0.00
GPU-batch + xcorr	0.088	0.111	0.199	325×	0.00
GPU-graph	0.086	0.134	0.220	295×	0.00

**Table 6:** Performances on RTX 3090Ti (median over 5 repetitions).

Tier	STFT [s]	XCorr [s]	Total [s]	Speedup	Max|Δ|
CPU	48.356	14.217	62.450	1.0×	—
CPU-multicore	13.358	2.725	16.083	4.0×	0.00
GPU per-sweep	0.206	0.305	0.510	127×	0.00
GPU-batch	0.167	0.305	0.472	137×	0.00
GPU-graph	0.188	0.305	0.493	131×	0.00

**Table 7:** Performances on GTX 1050Ti (median over 5 repetitions).

Tier	STFT [s]	XCorr [s]	Total [s]	Speedup	Max|Δ|
CPU	69.897	15.328	85.374	1.0×	—
CPU-multicore	33.547	7.252	40.853	1.58×	0.00
GPU per-sweep	3.083	2.043	5.127	12.62×	0.00
GPU-batch	3.100	2.049	5.148	12.57×	0.00
GPU-graph	3.132	2.048	5.180	12.49×	0.00

**Table 8:** Cross-architecture comparison. GPUs’ performance compared versus common AMD Ryzen CPU reference.

	RTX PRO 6000	RTX 3090Ti	GTX 1050Ti
Architecture	Blackwell	Ampere	Pascal
SMs	188	84	6
Memory bandwidth	1792 GB/s	1008 GB/s	112 GB/s
L2 cache	134 MB	6 MB	1 MB
Auto batch size	*B* = 200	*B* = 38	*B* = 1
STFT time	0.088 s	0.167 s	3.083 s
XCorr time	0.133 s	0.305 s	2.043 s
Total	0.221 s	0.472 s	5.127 s
Per-sweep	1.1 ms	2.4 ms	25.6 ms
Speedup	293×	137×	12.6×

**Table 9:** Runtime, per-stage timings, CPU-relative speed-up, and incremental gains are reported for successive GPU implementation variants, using a common AMD Ryzen CPU as reference.

Step	Total [s]	STFT [s]	XCorr [s]	CPU [x]	Step Δ
Baseline GPU	1.84	—	—	35×	—
+Data residency	0.525	0.366	0.160	123×	3.5×
+rfft xcorr	0.492	0.360	0.131	134×	1.07×
+Batched xcorr	0.471	0.360	0.111	137×	1.04×
+Combined remap	0.435	0.324	0.111	149×	1.08×
+float32 STFT	0.241	0.130	0.111	268×	1.81×
+Fused fiber selection	0.199	0.088	0.111	325×	1.21×

**Table 10:** Estimated maximum fiber coverage on RTX PRO 6000 Blackwell. The estimates are based on the three-parameter empirical model of Section 5.6 and should be read as indicative; the typical uncertainty is approximately ±25–30% when extrapolated to GPUs outside the training set.

Target Rate [Hz]	Time [ms]	Max Fiber Points	Fiber Length [m]
100	10	17,242	569
200	5	8224	271
500	2	2814	93
1000	1	1010	33

## Data Availability

The experimental OVNA dataset analyzed in this study was originally reported in Ref. [6]. The benchmark data, profiling results, mixed-precision validation outputs, and source code supporting the findings of this study are available from the corresponding author upon reasonable request.

## Abbreviations

The following abbreviations are used in this manuscript:

OVNA	Optical Vector Network Analyzer
DOFS	Distributed Optical Fiber Sensors
STFT	Short-Time Fourier transform
OFDR	Optical Frequency-Domain Reflectometry
FFT	Fast Fourier Transform
GPU	Graphical Processing Unit
FUT	Fiber Under Test
VNA	Vector Network Analyzer
